# Pilot Whales Attracted to Killer Whale Sounds: Acoustically-Mediated Interspecific Interactions in Cetaceans

**DOI:** 10.1371/journal.pone.0052201

**Published:** 2012-12-26

**Authors:** Charlotte Curé, Ricardo Antunes, Filipa Samarra, Ana Catarina Alves, Fleur Visser, Petter H. Kvadsheim, Patrick J. O. Miller

**Affiliations:** 1 Sea Mammal Research Unit, School of Biology, University of St. Andrews, Fife, St. Andrews, United Kingdom; 2 Netherlands Organization for Applied Scientific Research (TNO), The Hague, The Netherlands; 3 Kelp Marine Research, Hoorn, The Netherlands; 4 Norwegian Defense Research Establishment (FFI), Horten, Norway; Institut Pluridisciplinaire Hubert Curien, France

## Abstract

In cetaceans’ communities, interactions between individuals of different species are often observed in the wild. Yet, due to methodological and technical challenges very little is known about the mediation of these interactions and their effect on cetaceans’ behavior. Killer whales (*Orcinus orca*) are a highly vocal species and can be both food competitors and potential predators of many other cetaceans. Thus, the interception of their vocalizations by unintended cetacean receivers may be particularly important in mediating interspecific interactions. To address this hypothesis, we conducted playbacks of killer whale vocalizations recorded during herring-feeding activity to free-ranging long-finned pilot whales (*Globicephala melas*). Using a multi-sensor tag, we were able to track the whales and to monitor changes of their movements and social behavior in response to the playbacks. We demonstrated that the playback of killer whale sounds to pilot whales induced a clear increase in group size and a strong attraction of the animals towards the sound source. These findings provide the first experimental evidence that the interception of heterospecific vocalizations can mediate interactions between different cetacean species in previously unrecognized ways.

## Introduction

The last decade has seen a growing recognition of the importance of individual traits and behavior in the understanding of biological communities’ structure, moving away from describing ecological networks based on species-averaged data to start exploring patterns based on individuals [1]–[3]. In cetaceans, although various observations have reported interspecific encounters, very little is known on how these animals respond when they detect the presence of another cetacean species and how these interactions are mediated. For animals that rely mostly on vocal-auditory channels to communicate such as cetaceans, the interception of heterospecific vocalizations may inform non-intended receivers about the presence of predators, prey or competitors, enabling eavesdroppers to make adjustments of their behavior [4], [5]. Playback of natural sounds is the most relevant method to study the role of vocalizations in the interactions between animals [6]. However, this approach has been rarely attempted in wild cetaceans due mainly to the difficulty to reliably monitor the behavioral responses of these animals at sea [7]–[13]. Killer whales (*Orcinus orca*) can be both food competitors and potential predators of many other cetaceans [14]. As killer whales are a highly vocal species [15]–[17], we hypothesized that the interception of their vocalizations by other cetaceans is instrumental in mediating interspecific interactions.

We conducted our study in the Norwegian Sea where long-finned pilot whales (*Globicephala melas*) and killer whales live in sympatry [18]. In this area, killer whales have a food preference for Atlantic herring (*Clupea harengus*) [17], [19] although it is unknown if this is their only prey [20], [21]. Long-finned pilot whales feed primarily upon squid and eat occasionally small fish such as mackerel (*Scomber scombrus*) and herring [22]. Interactions between killer whales and pilot whales may be thus complex, including competitions for food resources and potentially anti-predator behaviors. Pilot whales hear well at the frequencies of killer whale vocalizations [23] and thus may assess and respond to killer whale presence by eavesdropping on their vocalizations. We conducted playback of herring-feeding killer whale sounds to long-finned pilot whales and we monitored the behavioral responses of the animals using an advanced high-resolution multi-sensor tag (D-tag) [24]. Using the radio beacon on this tag, we were able to track the position of the whale and thus (i) to quantify changes of horizontal movements and (ii) to monitor inter-animal spacing of the tagged whale’s group. To control for reactions to any unspecific acoustic stimulus, a broadband noise was played back to several subjects as a negative control. We expected that the animals would not react to this control or that they would react differently compared to the killer whale sounds stimulus.

## Materials and Methods

### Ethics Statement

Animal experiments were carried out with permission from the Norwegian Animal Research Authority (Permit No. 2004/20607 and S-2007/61201). Protocols were approved by the Animal Welfare and Ethics Committee of the University of St Andrews (AWEC, UK) and the Institutional Animal Care and Use Committee (IACUC) of the Woods Hole Oceanographic Institute (WHOI, USA).

### Acoustic Stimuli and Playback Procedure

We conducted our study in the Norwegian Sea aboard a research vessel in May/June 2008–2010. A zodiac boat was deployed from the vessel for tagging operations and playback experiments.

The killer whale sounds (KW) were previously recorded in Vestfjord, Norway, using D-tags and correspond to calls, clicks and tail slaps produced during herring-eating activity. Indeed, herring-feeding killer whales living in Norway exhibit a specific foraging behavioral pattern associated to the production of sounds that has been well-characterized [19]. Sound sections that were not part of the killer whale vocalizations, e.g., flow noise (due to swimming speed) and surfacing noise (breathing), were suppressed from the stimuli.

Control (CTRL) stimuli corresponded to sequences of silence during killer whale sound recordings, amplified to get an average root mean square power equal to the KW stimuli. These CTRL stimuli (0.5–10 kHz) have most energy distributed between 1 and 2 kHz, which correspond to the fundamental frequency of the majority of killer whale calls [25], [26].

For each stimulus type (KW and CTRL) 3 stimulus versions, i.e. collected from different acoustic recordings, were used among the tested whales to avoid pseudoreplication [27].

Sounds were generated using a M-Audio Microtrack II recorder and amplified by a Cadence Z8000 amplifier connected to a Lubell LL9642T underwater loudspeaker (frequency response: 0.2–20 kHz) submerged at a depth of 8 m. To later measure the sound level of the source and to ensure that sounds were faithfully played back by the system without distortion, playback stimuli were monitored using a calibrated hydrophone (Bruel & Kjaer 8105 amplified by a Bruel & Kjaer 2635 charge amplifier) placed at 1 m from the source and recorded using a M-Audio Microtrack II recorder. The sound level of the killer whale sounds composing the stimuli ranged from 140 to 155 dBrms re 1µPa (mean ± SD: 149±4 dBrms re 1µPa, N = 3 stimuli) which corresponds to the source level of killer whale vocalizations observed in natural conditions [28]. The sound level of control stimuli ranged from 145 to 150 dBrms re 1µPa (mean ± SD: 147±2 dBrms re 1µPa, N = 3 stimuli).

We tested 6 long-finned pilot whale groups (1 tagged whale per group) encountered inside the Vestfjord basin, Norway. Three whales were tested with KW, 2 whales with both CTRL and KW, and one whale was tested only with CTRL because of premature tag detachment. Each stimulus lasted 15 min and was played back twice. The average duration of the killer whale sounds within each 15 min KW stimulus was 11 min 2 sec ±35 sec (mean ± SD, N = 3). A recovery period of 10 min separated the different playback trials performed to a tested whale. At the start of playbacks, the sound source was positioned to the side of the tagged whale’s path, at a distance of 2400±943 m (mean ± SEM).

### Quantification of Changes in Horizontal Movements and Group Size

To monitor the behavioral responses of the animals, a D-tag [24] was non-invasively attached to the focal animal with suction cups at least 2 h preceding the start of playback. Time for tag release was programmed beforehand and the tag was recovered at the end of experiments. Aided by the radio beacon on the tag, we were able to visually track the positions of the animal at each surfacing. Positions of the surfacing tagged whale (range and bearing relative to the vessel heading, see Supplementary Material S1 for protocol details) (for N = 6 tagged whales) and group size defined as the number of subjects within 200 m of the tagged animal (for N = 4 tagged whales), were simultaneously recorded from the vessel at intervals of 3±2 min (mean ± SD) [29]. Baseline behavior was collected for a minimum of 1 h preceding the playbacks. For each playback, (i) we assessed whether the acoustic stimuli induced a change in the tagged whale’s group size and (ii) we measured a reaction score that was defined to quantify the attraction (positive score) or avoidance (negative score) of the tagged animal to the sound source (see Supplementary Material S1 for details on the reaction score and group size analyses; Fig. S1).

### Statistics

Each whale was exposed to several stimuli so to account for repeated measures we used Generalized Estimating Equation (GEE) models [30] to test whether the stimulus type and playback order (independent variables) had an influence on the response (dependent variable), i.e. reaction score or change in group size. As the Sandwich variance estimator can be biased for small numbers of clusters, a Jackknife variance estimator was applied.

## Results

**Figure 1:**
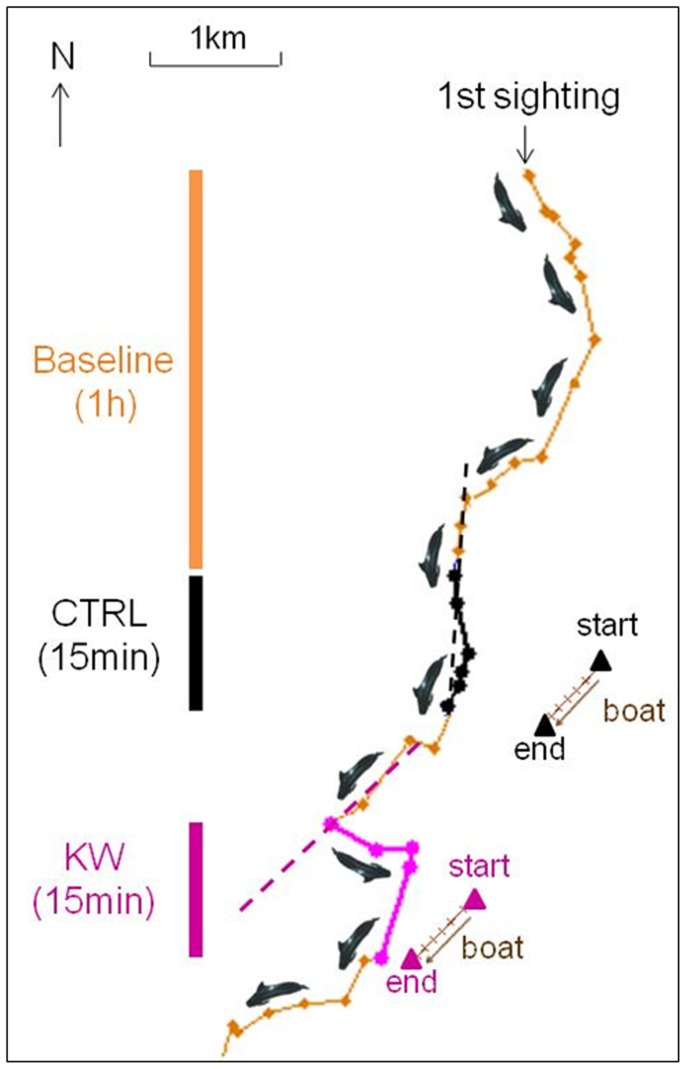
Track of tagged whale gm10_158d. Each dot corresponds to 1 sighting. Orange: baseline period. Black: control playback (CTRL) period. Magenta: killer whale playback (KW) period. Triangles: position of the sound source, at start and end of playbacks. Dotted lines: projected course of the whale as if the animal had kept its initial direction of horizontal movement.

**Figure 2:**
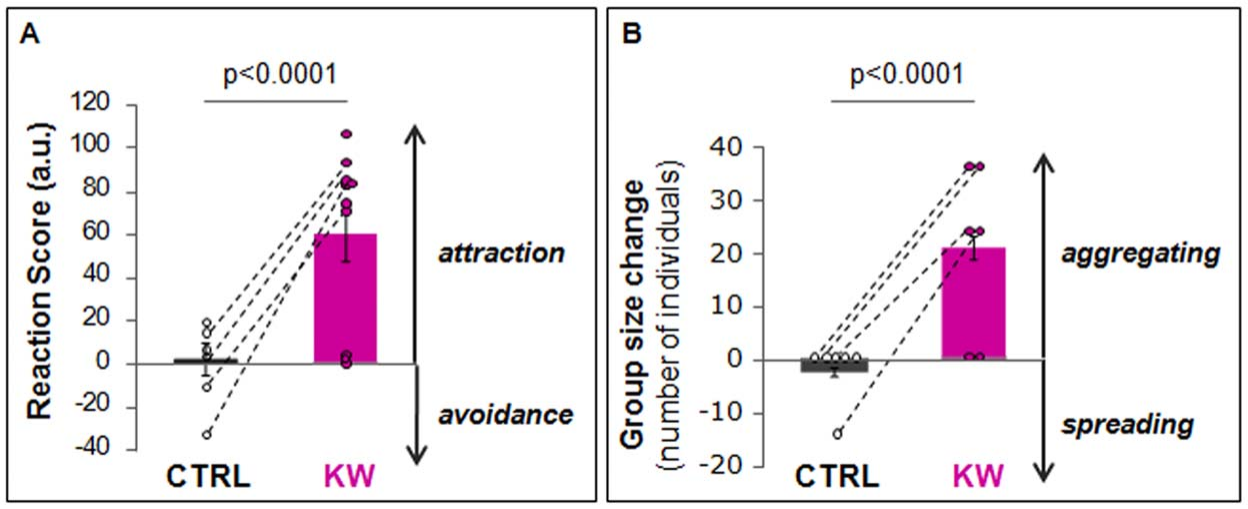
Response of tagged long-finned pilot whales to KW and CTRL playbacks. (A) Reaction scores for KW playbacks (N = 5 whales tested twice, n = 10 trials) and CTRL playbacks (N = 3 whales tested twice, n = 6 trials). Positive values: attraction towards the sound source; negative values: avoidance. Dashed lines link the reaction scores of the same subject. (B) Change of group size during KW and CTRL playbacks (for each stimulus type: N = 3 individuals tested twice, n = 6 trials). Positive values: whales aggregating around tagged animal; negative values: whales spreading. Error bars give mean ± SEM.

The whales tested with control playbacks kept travelling broadly in the same direction they were travelling before the start of playback (Fig. 1), resulting in a mean reaction score close to zero (Fig. 2A). In contrast, 4 out of the 5 whales tested with killer whale sounds made a clear turn, changing their course towards the playback speaker (Fig. 1). The positive mean reaction score of the killer whale playbacks (Fig. 2A) was significantly different from that of the controls (GEE, *P*<0.0001, Table S1). Results of the GEE (Generalized Estimating Equation) models indicated a strong significant effect of stimulus type on both reaction score and group size but no order effect (Table S1). For the movement reaction score, after a Jackknife variance estimator, the stimulus type effect was still strongly significant whereas an order effect appeared but remained low. For the group size data, more samples would be necessary to apply the Jackknife variance estimator.

This attraction to the sound source was not only observed for the tagged whale but also for many other individuals in the area, resulting in a significant increase in group size (Fig. 2B) (GEE, *P*<0.0001, Table S1), and a general movement of whales towards the playback speaker.

**Figure 3:**
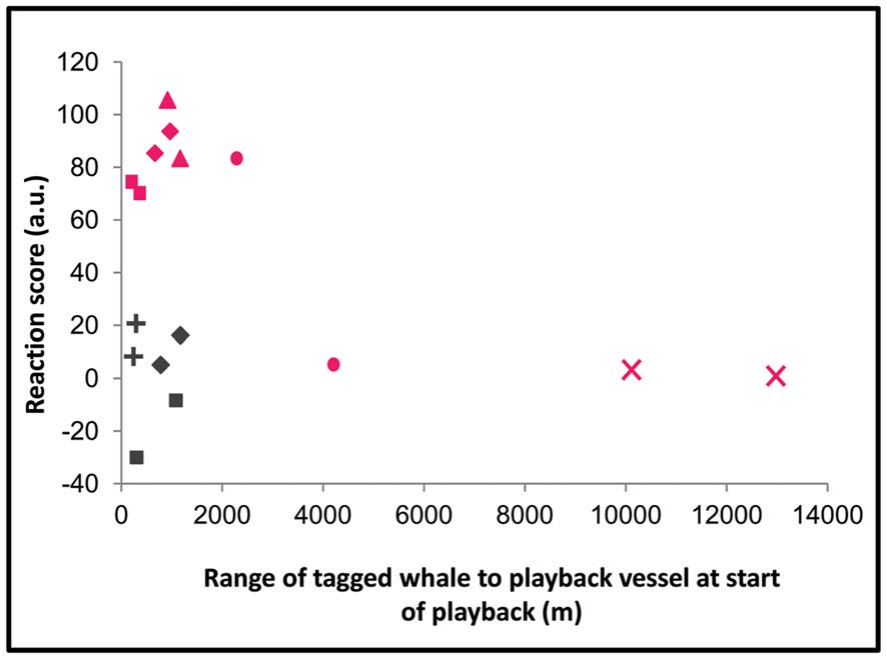
Reaction scores for the 10 KW playback trials (magenta) and the 6 CTRL playback trials (grey) versus the distance between tagged whale and sound source at start of playback. The 6 different signs represent the 6 tagged whales (▵◊□○X+).

For the tagged whale that never responded to the KW sounds (reaction score close to zero for both KW trials), the playback was conducted at a much higher distance to the whale (>10 km) compared to the 4 other tested animals (Fig. 3). Moreover, among the 4 whales that did respond to the KW sounds playback, one tagged whale exposed to KW sounds at a range of 2.35 km did react (positive reaction score) but did not react to the second KW trial (reaction score close to zero) that was conducted at a longer range to the whale (4.27 km). It’s thus possible that for the 3 KW trials that showed no response, the whales were too far from the sound source (Fig. 3) to detect the sounds (see Supplementary Material S1 for estimation of the received sound pressure levels).

## Discussion

We showed that killer whale sounds recorded during herring-feeding activity clearly attracted pilot whales which provide the first experimental evidence of cetaceans’ attraction towards the vocalizations of another cetacean species. These findings demonstrated that long-finned pilot whales adjusted their movement path and social behavior when they detected killer whale vocalizations. These results represent a unique behavioral response compared to previous studies [6], [12], [13]. Indeed, pioneering studies conducted 40 years ago reported grey whales and belugas’ avoidance in response to the playback of fish-eating killer whale sounds [12], [13]. Moreover, a recent study showed that a beaked whale responded with avoidance to the playback of mammal-eating killer whale sounds [7]. It thus seems that both types of killer whale vocalizations i.e., mammal or fish-eating sounds, have elicited an avoidance response. However, it can be pointed out that all these sounds tested so far were unfamiliar. As cetaceans have probably the ability to learn to associate sounds to specific contexts (e.g. associating mammal-eating killer whale sounds to a threat) like it was demonstrated on seals [31], one could expect that familiar orca sounds would have lead to other reactions in the tested animals.

Here, the attraction of long-finned pilot whales towards local herring-feeding killer whale sounds source is consistent with visual observations reported from the Norwegian Sea and from the strait of Gibraltar where long-finned pilot whales have been seen approaching and chasing respectively herring- and tuna-feeding killer whales [26], [32], [33]. Killer whales have been observed fleeing away from pilot whales which represent a unique case of killer whales avoiding another cetacean species.

One possible explanation to the approach reaction of pilot whales to herring-feeding killer whale sounds playbacks could be an attraction to a location of food being predated upon by a competitor, as the killer whale sounds were recorded during feeding upon herring. Pilot whales may have been drawn to the killer whale sounds as a perceived opportunity for feeding on the same forage species. Indeed, pilot whales off Norway feed mostly on squid but do take also schooling fishes such as herring [22] which is the main diet of killer whales in this area [17], [19]. Alternatively, the response may be a mobbing strategy whereby individuals group together and move towards killer whales, either as an anti-predator strategy or as an aggressive behavior towards heterospecifics. In that case, the fact that pilot whales respond so strongly to vocalizations of familiar fish-eating killer whales that likely pose no threat to them suggest that long-finned pilot whales exhibit a template of harassment response regardless of killer whales’ prey preferences and did not habituate to these particular vocalizations. The specificity of this response can be further explored by conducting playbacks with different marine mammals’ sounds (e.g. unfamiliar sounds, different prey-eating and non-feeding sounds) like it has been explored on seals [31].

In conclusion, our study demonstrates that the outcomes of interactions between cetacean species at the individual scale is an important factor in driving the sociality and ranging patterns of these animals which may influence, at a higher scale, the dynamics of cetaceans’ communities. These results open novel applications in conservation biology since playbacks could be wisely used as a non-invasive method in rescue operations of cetaceans at risk of stranding. On the other hand, the discovery of attractive signals for cetaceans raises the issue of exploitation by whale watching and hunting companies.

## Supporting Information

Figure S1 **Distances between tagged whale gm10_158d and the sound source during CTRL playback (left) and KW playback (right) experiments.** Dotted lines: distances obtained projecting movement based upon sightings in the 10 min-period prior to the start of each playback. Solid lines: actual distances. rs: reaction score, defining as the difference between distance at the last projected sighting and the distance at the last actual sighting.
(TIF)

Table S1 **Results of the GEE models on reaction score and change of group size, with both independent variables: playback order and stimulus type.** Shown are estimates, their standard errors (s.d.), and p-values (before and after Jackknife estimator).
(DOC)

Material S1 **Protocol details, method for reaction score and group size analyses and estimation of the sound pressure levels received by the whales.**
(DOC)
