# Supplementary figures and images for: Pilot Whales Attracted to Killer Whale Sounds: Acoustically-Mediated Interspecific Interactions in Cetaceans
Source: PLoS One. 2012 Dec 26;7(12):e52201. doi: 10.1371/journal.pone.0052201 (PMC3530591; doi:10.1371/journal.pone.0052201)

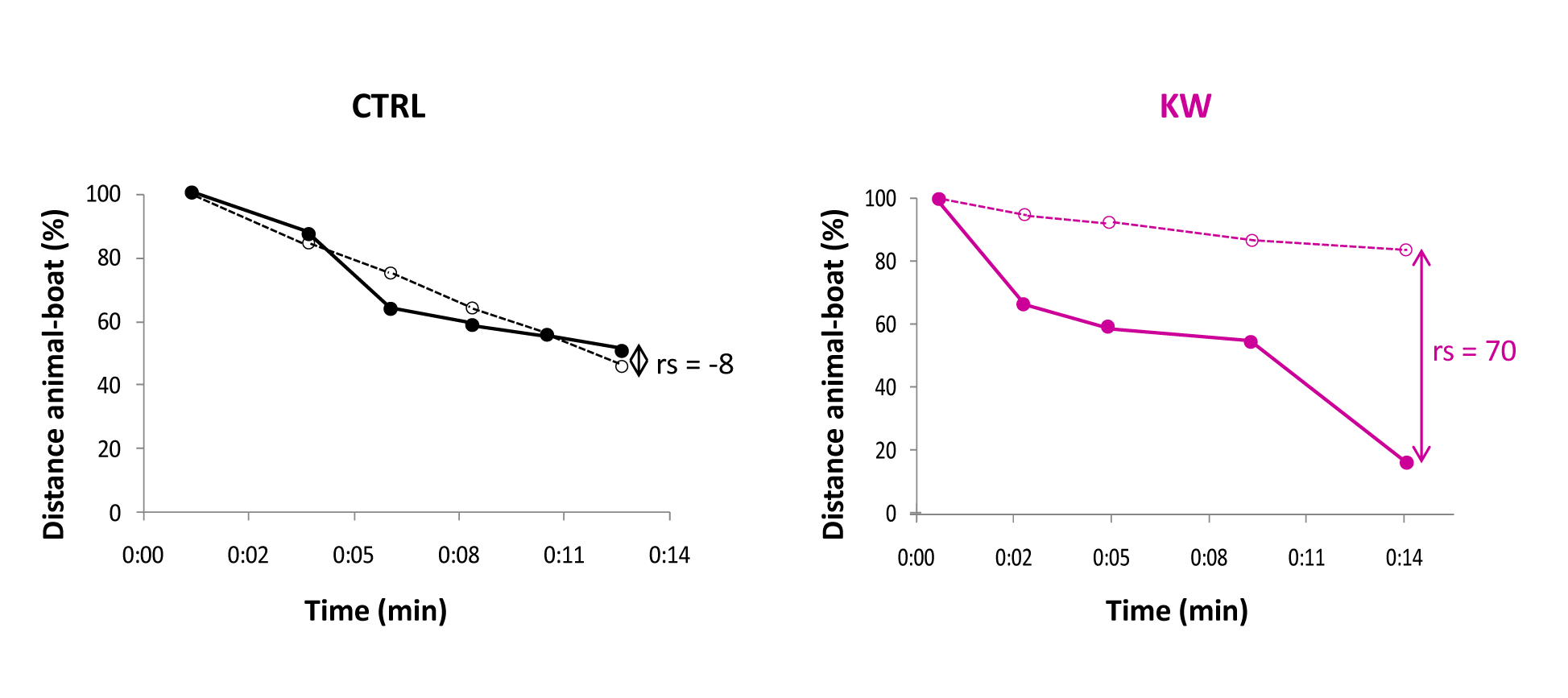

Supplement: Figure S1 — Distances between tagged whale gm10_158d and the sound source during CTRL playback (left) and KW playback (right) experiments. Dotted lines: distances obtained projecting movement based upon sightings in the 10 min-period prior to the start of each playback. Solid lines: actual distances. rs: reaction score, defining as the difference between distance at the last projected sighting and the distance at the last actual sighting. (TIF) [file pone.0052201.s001.tif]
