# Supplementary material for: Pilot Whales Attracted to Killer Whale Sounds: Acoustically-Mediated Interspecific Interactions in Cetaceans
Source: PLoS One. 2012 Dec 26;7(12):e52201. doi: 10.1371/journal.pone.0052201 (PMC3530591; doi:10.1371/journal.pone.0052201)
Supplement: Table S1 — Results of the GEE models on reaction score and change of group size, with both independent variables: playback order and stimulus type. Shown are estimates, their standard errors (s.d.), and p-values (before and after Jackknife estimator). (DOC) [file pone.0052201.s002.doc]

**Supplementary Table S1:**

| Dependent variable of the GEE model; sample size |  | Estimate ± s.d.;  p-value (Sandwich variance estimator) | Estimate ± s.d.;  p-value (Jackknife variance estimator) |
| --- | --- | --- | --- |
| Reaction score;  N=6 whales | Intercept | 45.20 ± 31.54 | 45.20 ± 15.21 |
|  | Order | 9.33 ± 10.25; p=0.36 | 9.33 ± 4.54; p=0.04 |
|  | Stimulus type | -68.44 ± 19.40; p=0.0004 | -68.44 ± 8.96; p<0.0001 |
| Change in group size; N=4 whales | Intercept | 22.98 ± 13.89 | sample size insufficient (N=4 whales) to apply Jackknife variance estimator. |
|  | Order | 0.79 ± 2.09; p=0.7045 |  |
|  | Stimulus type | -32.53 ± 5.44; p<0.0001 |  |
