# Supplementary material for: Pilot Whales Attracted to Killer Whale Sounds: Acoustically-Mediated Interspecific Interactions in Cetaceans
Source: PLoS One. 2012 Dec 26;7(12):e52201. doi: 10.1371/journal.pone.0052201 (PMC3530591; doi:10.1371/journal.pone.0052201)
Supplement: Material S1 — Protocol details, method for reaction score and group size analyses and estimation of the sound pressure levels received by the whales. (DOC) [file pone.0052201.s003.doc]

**Supplementary Material S1**

***Protocol details***

The D-tags we used in this study included hydrophones, 3-axis accelerometers, 3-axis magnetometers and depth sensors. For the present work, we only used the radio beacon mounted on the tag to visually track the positions of the tagged whale.

At each surfacing of the tagged whale, we measured the distance from the vessel to the whale, the bearing to the whale relative to the vessel heading, and the vessel magnetic or true heading (see details in Supplementary Reference S1). Distance was measured using laser-range finders and relative bearing to the whale was measured using a protractor with a pointer. At each position recorded, the vessel heading was measured with a Seagate fluxgate compass or by course over ground measured with a GPS. For each recorded position of the tagged whale, we simultaneously scored the group size defined as the number of subjects within 200 m of the focal animal (for N=4 tagged whales). Visual data collection including the tagged whale position and group size was recorded using Logger software made available by the International Fund for Animal Welfare.

***Reaction score and group size analyses***

A reaction score was defined to quantify the attraction/avoidance of tagged animals to the playback sounds source. For each playback, we projected the whale’s course as if the animal had kept its initial direction of horizontal movement, *i.e*., direction based on the baseline sightings of the 10 min-period preceding the start of playback. The distance between animal and sound source (D) was measured for the actual and the projected sightings and normalized to the initial D (see Fig. S1). The reaction score is the difference between D at the last projected sighting and D at the last actual sighting.

Moreover, for each playback, we determined the change of group size by calculating the difference of maximum group size between the playback period and the 10 min period preceding the playback.

***Estimation of the received sound pressure levels***

Knowing the sound pressure level of the acoustic stimuli (SL) that we measured at 1m from the source and the distance between the sound source and the whale (D) at the start of the playback, we were able to estimate the sound pressure level received by the whale (RL). In the environment where the experiment were conducted, the transmission loss (TL) increased roughly with 18log_10_ (D) (Supplementary Reference S2). The RL was estimated using the following formula: RL = SL – 18log_10_ (D). The estimated RL ranged from 84 to 103 dB for the 7 trials for which the whales did respond to the KW playbacks and from 70 to 79 dB for the 3 non response KW playback trials. Pacini et al. (2010) measured the hearing thresholds on one captive long-finned pilot whale for frequencies between 4 and 100 kHz. They found that the thresholds ranged from 65 to 77 dB for frequencies between 4 and 20 kHz in a quiet environment. KW calls have most energy distributed between 1 and 2 kHz, but there is no data so far on the hearing thresholds of long-finned pilot whales for frequencies below 4 kHz. It is therefore possible that in our study, the KW playback sounds were masked in the ambient noise and so that the non responding whales did not hear the sounds.

***Supplementary References:***

S1. Miller PJO, Antunes R, Alves AC, Kuningas S, Curé C, Visser F, White P, Aoki K (2010) 3S-2010 Baseline Cruise Report: baseline behavior of long-finned pilot whales and sperm whales.

S2. Wensveen, PJ (2012) Effects of sound propagation and avoidance behaviour on naval sonar levels received by cetaceans. Master Thesis, University of St-Andrews. Available: http://hdl.handle.net/10023/3194
